# Primary Technology-Enhanced Care for Hypertension Scaling Program: Trial-Based Economic Evaluation Examining Effectiveness and Cost-Effectiveness Using Real-World Data in Singapore

**DOI:** 10.2196/59275

**Published:** 2025-04-15

**Authors:** Yi Wang, Shilpa Tyagi, David Wei Liang Ng, Valerie Hui Ying Teo, David Kok, Dennis Foo, Gerald Choon-Huat Koh

**Affiliations:** 1 Saw Swee Hock School of Public Health National University of Singapore and National University Health System Singapore Singapore; 2 Future Primary Care Ministry Of Health Office for Healthcare Transformation Singapore Singapore; 3 National Healthcare Group Polyclinics Singapore Singapore

**Keywords:** telehealth monitoring, hypertension, cost-effectiveness analysis, real-world data, Singapore, telehealth, cost-effectiveness, blood pressure monitoring, health care, teleconsultation, chatbot, regression analysis, medical cost

## Abstract

**Background:**

Telehealth interventions are effective in hypertension management. However, the cost-effectiveness of using them for managing patients with hypertension remains inconclusive. Further research is required to understand the effectiveness and cost-effectiveness in the real-world setting.

**Objective:**

The Primary Technology-Enhanced Care for Hypertension (PTEC-HT) scaling program, a telehealth intervention for hypertension management, is currently being scaled nationwide in Singapore. The program comprises remote blood pressure (BP) monitoring at home, health care team support through teleconsultations, and in-app support with a digital chatbot. This study aimed to evaluate the program’s effectiveness and cost-effectiveness.

**Methods:**

For patients under the PTEC-HT scaling program, BP readings over 6 months and 12 months, age, and gender were collected within the program. Health care use, health care cost, and patient ethnicity were extracted from the National Healthcare Group Polyclinics. For patients in the usual care group, demographic information, clinical data, health care use, and health care costs were extracted from the national claims records. Comparing the PTEC-HT scaling program with usual care, a trial-based economic evaluation using patient-level data was conducted to examine the effectiveness and cost-effectiveness over time horizons of 6 months and 12 months. The health care system’s perspective was adopted. Regression analysis and exact matching were used to control for the differences between the PTEC-HT group and the usual care group.

**Results:**

For the 6-month analysis, 427 patients were included in the PTEC-HT group, and 64,679 patients were included in the usual care group. For the 12-month analysis, 338 patients were included in the PTEC-HT group, and 7324 patients were included in the usual care group. Using exact matching plus regression, in the 6-month analysis, the probability of having controlled BP was 13.5% (95% CI 6.3%-20.7%) higher for the PTEC-HT group compared to the usual care group. In the 12-month analysis, the probability of having controlled BP was 16% (95% CI 10.7%-21.3%) higher for the PTEC-HT group. Without considering the cost of the BP machine and program maintenance cost, the direct medical cost was S $57.7 (95% CI 54.4-61.0; a currency exchange rate of S $1=US $0.74 was applicable;) lower per patient for the PTEC-HT group in the 6-month analysis and S $170.9 (95% CI 151.9-189.9) lower per patient for the PTEC-HT group in the 12-month analysis. With the cost of the BP machine and program maintenance considered, compared to usual care, the PTEC-HT program reached breakeven at around the sixth month and saved S $52.6 (95% CI 33.6-71.6) per patient at the 12th month.

**Conclusions:**

Implemented in a real-world setting in Singapore, our study showed that the PTEC-HT scaling program is more effective in controlling BP status with lower cost compared to the usual care over 12 months.

## Introduction

With improved adoption of personal devices and better infrastructure for internet access, telehealth has gained popularity and become more relevant in assisting people to manage their health. Telehealth enables health care delivery from a distance using information communication technologies [1]. Telehealth can potentially relax health care institution capacities, provide more convenient health care services, and promote better health management [1-3].

The effectiveness of telehealth interventions in hypertension management has been established in the literature [4,5]. A recent systematic review and meta-analysis of randomized controlled trials reported that using mobile app-assisted self-care interventions was associated with significant reductions in both systolic (standardized mean difference −0.17, *P*=.02) and diastolic (standardized mean difference −0.17, *P*=.02) blood pressure (BP) [4]. Another review reported a reduction in both systolic and diastolic BP of 3.78 mm Hg and 1.57 mm Hg, respectively, irrespective of differences in frequencies of reminders, interactive patterns, intervention functions, and duration [5]. Moreover, such interventions are reported to be relatively more effective among patients with more severe hypertension at baseline [6].

Based on a systematic review synthesizing evidence on the cost-effectiveness of telemedicine in the management of cardiovascular diseases (inclusive of hypertension), telemedicine was reported to be cost-effective when used concurrently with the usual care [7]. However, the cost-effectiveness results were not stratified by different cardiovascular diseases and associated risk factors. The economic evaluation evidence of telehealth interventions for hypertension management was inconclusive [5], thus highlighting the need for further research. Additionally, this evidence has been limited to trial settings, with little to no evidence from real-world implementation settings. With differences in the outcomes and generalizability implications across these 2 settings being well-established [8], our study would be a timely addition to existing literature.

The Primary Technology-Enhanced Care for Hypertension (PTEC-HT) program includes core components of telemonitoring of BP, telesupport from the care team, and teletitration of medications, where indicated based on developed clinical protocols. The development of the PTEC-HT program was evidence-based and implemented telehealth components with proven effectiveness, such as the logging of BP readings, automated feedback to the patients on submitted readings, regular reminders, and providing the ability to visualize submitted data and education materials [4].

The Ministry of Health Office for Healthcare Transformation (MOHT) is an agile unit focused on adopting an experimental and evidence-based approach to redesigning health care in Singapore by providing effective new end-to-end system-level solutions [9]. MOHT conducted a pilot trial involving 120 patients from a public primary care clinic in Singapore to evaluate the effectiveness of the PTEC-HT program in a controlled setting adopting a quasi-experimental design. After a follow-up of 6 months, the PTEC-HT program was effective in reducing the BP as well as cost-effective as compared to usual care. Additionally, greater satisfaction was reported for the PTEC-HT group as compared to the usual care group [10]. Based on the success of the PTEC-HT pilot trial [10], MOHT embarked on the implementation of the PTEC-HT program in the real-world setting, specifically across the entire public primary care system over 5 years. This is the first nationwide initiative of scaling a telehealth intervention for the management of patients with hypertension and hence presents a unique opportunity to examine the effectiveness and cost-effectiveness of this previously successful pilot in a real-world setting. Henceforth, the “PTEC-HT scaling program” would be used to refer to the PTEC-HT program in the scaling phase being implemented in the real-world setting (as opposed to the PTEC-HT program in the pilot phase).

The objective of this study is to evaluate the effectiveness and cost-effectiveness of the PTEC-HT program. Focusing on patients with hypertension who visited the public primary care clinics in Singapore, this study examined the BP of patients and the cost of managing patients in the real-world setting over 6 months and 12 months.

## Methods

### Overview

The reporting follows the CHEERS (Consolidated Health Economic Evaluation Reporting Standards) 2022 [11]. A completed CHEERS checklist has been included in Multimedia Appendix 1.

### Health Economic Analysis Plan

The health economic analysis plan was developed to ensure the results are comparable to the economic evaluation of the PTEC-HT program in the pilot phase. Clinicians’ input was sought on inclusion and exclusion criteria for identifying patients in the usual care group. Econometric methods were then identified to address the potential bias that arises from analyzing real-world data. The analysis plan was communicated to stakeholders before starting the analysis.

### Description of PTEC-HT Scaling Program

**Figure 1:**
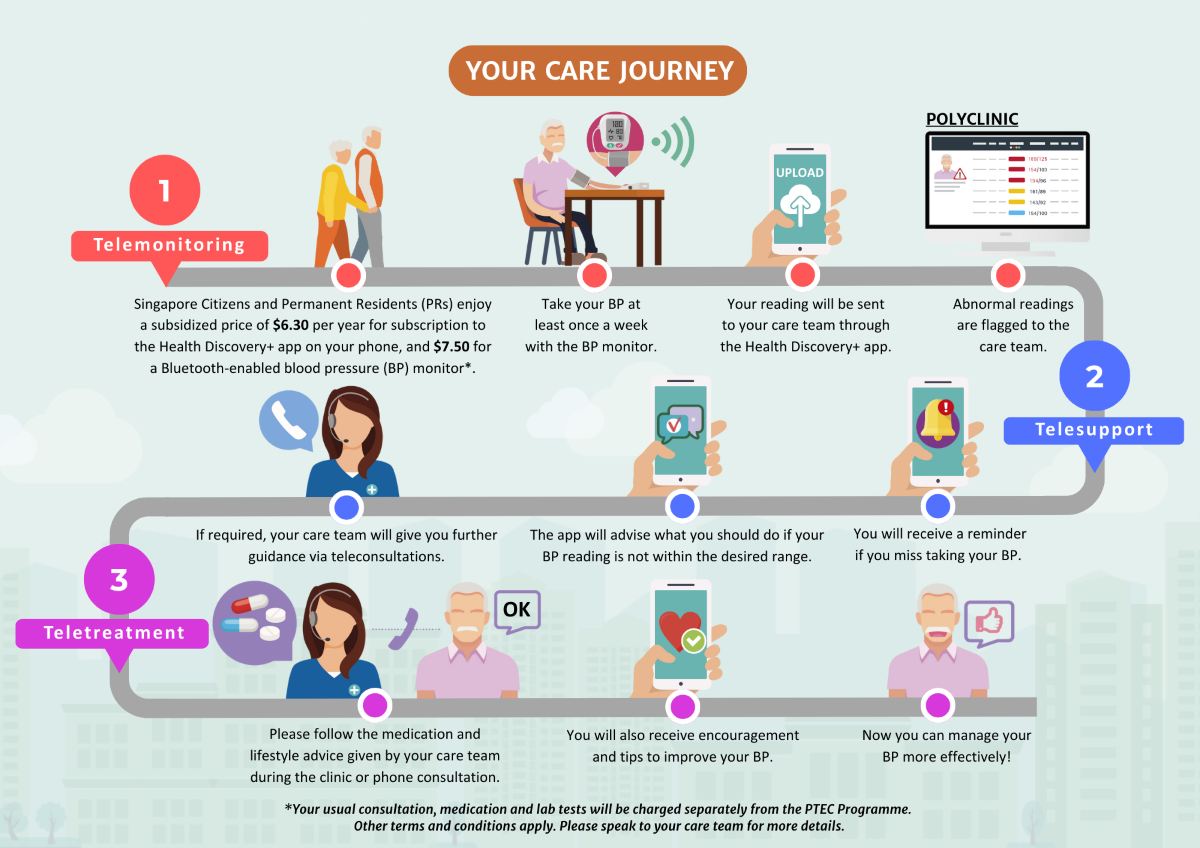
Care journey of enrolled patients on the PTEC-HT scaling program. BP: blood pressure; PTEC: Primary Technology-Enhanced Care; PTEC-HT: Primary Technology-Enhanced Care for Hypertension.

The PTEC-HT scaling program is the first nationwide intervention under the Primary Technology-Enhanced Care (PTEC) initiative, which aims to empower patients with high BP to self-manage their condition with remote support from their health care team in the comfort of their home, with the help of simple technology. It is built on the national IT platform of Smart Health Vital Signs Monitoring provided by the Integrated Health Information Systems, the health technology agency for Singapore [12]. The program comprises the following three components: (1) remote monitoring of BP with a Bluetooth-enabled BP machine at least once a week with the readings transmitted to the public primary care clinic through the Health Discovery+ app; (2) care team support including monitoring of transmitted readings every month, contacting the patient via teleconsultations if their condition is not well-controlled or needs medication titration; and (3) in-app support with the provision of digital chatbot through helpful advice and reminders [12]. The detailed workflow or the care journey of enrolled patients on the PTEC-HT scaling program is illustrated in Figure 1.

The PTEC-HT program is a nurse-driven model designed to minimize physician involvement by empowering nurses to manage most BP-related alerts and patient issues independently using detailed clinical protocols. Nurses are trained and provided with comprehensive protocols to handle most clinical alerts without requiring frequent physician input. This approach significantly reduces the need for coconsults and supports the program’s aim to optimize resource use. The time required for doctor consultation is minimal in this model and occurs only when necessary, such as in complex clinical scenarios or decisions requiring medical expertise (eg, adjusting treatment plans or determining the need for an in-clinic visit). For medication titration, physicians often provide standing instructions, enabling nurses to manage follow-up teleconsultations independently. This streamlined workflow avoids unnecessary delays and minimizes physician workload. Similarly, technical matters are addressed through a dedicated IT support team. Nurses are not typically involved in resolving complex technical issues beyond their expertise, ensuring their clinical focus remains uninterrupted.

### Study Design

Aligned with the pragmatic implementation setting of the PTEC-HT scaling program, we adopted a trial-based economic evaluation. The population comprised a subgroup of patients enrolled in the PTEC-HT scaling program (who completed at least 6 months or 12 months on the program at the time of conduct of this study) and a historical control or usual care group. This historical usual care group was assembled from the national claims records considering the limited funding and manpower constraints of this real-world evaluation of a nationwide implemented program as opposed to a pilot trial. The national claims record in Singapore “maintains an island-wide database of acute and outpatient healthcare service utilization and associated expenditures” [13]. For the PTEC-HT group, the eligible patients included those with enrollment anytime between the start of the PTEC-HT scaling program in August 2020 to January 31, 2021, from National Healthcare Group Polyclinics (NHGP) [14]. Additionally, patients in the PTEC-HT group had follow-ups of at least 6 months on the program. For both the PTEC-HT and usual care group, the patients were included if they met the following criteria: aged 21 years and older, having hypertension with or without hyperlipidemia. Patients were excluded if they had pre-existing complications, including prediabetes, diabetes mellitus, stroke, ischemic heart disease, nephritis, and nephrosis. The *ICD-10* (*International Classification of Diseases, Tenth Revision*) codes used for patient exclusion are presented in Multimedia Appendix 2. The usual care group was assembled based on pooled data from 2 waves of historical controls (or usual care group) spanning (1) January 2018 to December 2018 and (2) January 2019 to December 2019. Patients were included in the analysis if their BP measurements were available in the 1st month, 6th month, or 12th month. This mirrors patients’ selection in the PTEC-HT group as patients who complied with the required care were included in the analysis. The choice of this time period of follow-up of the usual care group was determined by the data availability in the national claims record and the related lag period for the refreshing or updating of the database.

The population, intervention, comparator, outcomes, perspective, and time horizon are summarized in Table 1. The cost was in 2020 Singapore dollars. The health care system perspective was used, following the recommendation by the Agency for Care Effectiveness Singapore [15]. The time horizon was selected based on the availability of health outcome measurements. Regression analyses were conducted to estimate the incremental health benefit and incremental cost. The Good Research Practice Task Force Report from the International Society for Pharmacoeconomics and Outcomes Research was referred to guide the analysis [16].

**Table 1:** Economic evaluation setup.

Population	Patients with hypertension without complication
Intervention	PTEC-HT^a^ program
Comparator	Usual care
Outcomes	BP^b^ status, incremental health outcome, direct medical cost, incremental cost
Perspective	Health care system
Time horizon	6 months and 12 months

^a^PTEC-HT: Primary Technology-Enhanced Care for Hypertension.

^b^BP: blood pressure.

### Data Collection—Cost and Health Outcomes

For the PTEC-HT scaling program group, the Health Discovery Plus (HD+) system [17] was used to extract the BP readings over 6 months and 12 months (from the registration date), age, gender, and enrollment date of the patient into the program. NHGP database was used to extract the use and cost data along with the ethnicity of the patients. The use and associated total cost were tagged as hypertension-related or not based on a list of service subcategory codes developed by the team of clinicians or health care providers in the NHGP cluster, who were also involved in the PTEC-HT pilot trial [10]. Additionally, the total direct medical cost was categorized into the following 4 categories: doctor consults, nurse consults, medication-related, and laboratory-related, following the methodology adopted for assessing cost-effectiveness in the PTEC-HT pilot to ensure comparability of findings across the pilot and scaling phase. For the teleservices provided by nurses, including reviewing BP measures and following up with patients through teleconsultation, the human capital approach was used to calculate the cost. Teleconsultations conducted by nurses were extracted from NHGP records. The exact duration of each teleconsultation was not captured under the PTEC-HT program. Drawing on a similar telemonitoring intervention for patients with type 2 diabetes mellitus, we assumed an average duration of 10 minutes per teleconsultation under PTEC-HT [18]. This duration includes identifying alerts, providing consultations, and arranging necessary follow-up actions. The standard salary of junior nurses was used to estimate the monetary value of time spent by nurses. Additional costs considered included a Bluetooth-enabled BP machine and program maintenance including patients’ data storage and app maintenance.

For the usual care group, both the pooled clinical data and use and associated total cost data were extracted from the national claims records. Similar to the PTEC-HT group, the use and associated total cost were tagged as hypertension-related or not, and the total cost was categorized into the above 4 categories. While doctor consults, nurse consults, and laboratory-related costs were tagged as hypertension-related versus not, for the medication-related costs, due to the unavailability of detailed, clean data in the national claims record at the time of the conduct of this study, this tagging was not possible. Hence, overall medication-related costs (including both hypertension related and unrelated) were included in the current analysis for both the PTEC-HT group and the usual care group.

For BP measurement, participants in the intervention group were trained by health care professionals at the start of the program to measure BP at home using validated automated devices, following internationally recognized guidelines, for example, seated position, rest before measurement, and multiple readings per session. This training ensured consistency and accuracy in home BP readings. While clinic-based BP measurement in the usual care group may not have been standardized or rigorously documented as is typical in real-world settings, in the Singapore public primary care setting, health care professionals are trained to conduct BP measuring in a clinic setting.

### Analytics

Summary statistics were presented for demographic information, BP status, and direct medical cost. BP status at month 1, month 6, month 7, and month 12 were examined. For patients with missing BP readings at month 1, month 6, month 7, and month 12, we used BP readings at month 2, month 5, month 8, and month 11, respectively, to proxy. Mean BP readings for each month were calculated for the patients. A patient was defined as having controlled BP in a month if the mean systolic BP was less than 140 mm Hg and the mean diastolic BP was less than 90 mm Hg. A patient was defined as having improved BP if both mean systolic BP and mean diastolic BP decreased.

For the usual care group, data were divided into 6 months and 12 months for the analysis. When examining the 6-month outcomes, the data of the usual care group was divided into 4 periods of 6 months, namely January 2018 to June 2018, July 2018 to December 2018, January 2019 to June 2019, and July 2019 to December 2019. The analyses were carried out by comparing the data of the intervention group to the pooled data over the four 6-months periods. When examining the 12-month outcomes, the data of the usual care group was divided into 2 periods of 12 months, namely January 2018 to December 2018 and January 2019 to December 2019. The analyses were carried out by comparing the data of the intervention group to the pooled data over the two 12-month periods.

The outcome variables in the regression analysis were BP status and direct medical cost. BP status is a dummy variable, with the value being 1 for controlled patients and 0 for uncontrolled patients. Logistic regression was used for BP status. Panel data structure was considered for repeated BP measurement over time using the population-average panel data model. BP status in the first month and BP status in the last month (sixth month or 12th month) were used as outcome variables. The method used to analyze BP status resembles the difference-in-differences method taking advantage of the panel data. The difference in the improvement in BP status between the PTEC-HT group and the usual care group was due to interest. The group-fixed effect was controlled, for example, the BP measuring setting.

Direct medical cost is a continuous variable. Generalized linear regression with gamma distribution and log link was used for examining direct medical costs. The direct medical cost was aggregated over 6 months for the 6-month analysis and aggregated over 12 months for the 12-month analysis. Due to the nonlinear regression equations, marginal effects were calculated to represent the differences, that is, incremental health benefit and incremental cost, between the PTEC-HT group and the usual care group.

Control variables included a dummy variable indicating the PTEC-HT group and usual care group, age (continuous variable), gender (female and male), and ethnicity (Chinese and non-Chinese). For the analysis of BP status, a dummy variable indicating at the beginning or the end of the intervention, and the interaction term between this variable and the dummy variable indicating PTEC-HT group and usual care group were included.

To further control for the differences between the intervention group and usual care group, and reduce the potential associated bias, matching was used [19]. Exact matching with replacement was selected among several matching method options because there were much more observations from the usual care group. Variables used for matching included BP status at the 1st month, age, gender, and ethnicity. For each participant in the PTEC-HT group, we identified the participants with the same age, gender, ethnicity, and BP status at the 1st month from the usual care group as the control. When several patients in the usual care group were matched to a patient in the PTEC-HT group, an equal weight was assigned to these patients. For example, if 5 patients from the usual care group were identified to match with a patient from the PTEC-HT group, each of the 5 patients from the usual care group received a weight of 0.2. The distributions of variables used for matching were identical between the PTEC-HT group and the usual care group following exact matching.

### Sensitivity Analysis

Additional analyses were conducted to examine the robustness of the results, including subgroup analysis, quantile regression, and regression analysis removing the top 2% of the cost. Details for the additional analysis are provided in Multimedia Appendix 2. All the statistical analyses were conducted using Stata (version 16; StataCorp LLC). The significance level was set at 5%.

### Ethical Considerations

#### Ethical Review and Approvals

This study involved a secondary analysis of existing, deidentified data and was conducted retrospectively. This study was approved by the National Healthcare Group Domain Specific Review Board (NHG DSRB reference: 2023/00347). As part of this approval, the institutional ethics board determined that the use of existing data without identifying individual patients did not require written informed consent, as this was a secondary analysis conducted per ethical guidelines.

#### Informed Consent and Waiver

The original data collection was conducted as part of a health care program, and written informed consent was obtained from patients during the initial enrollment into the program for the use of their data for health care purposes. For this secondary analysis, no additional informed consent was required as per the institutional ethics board’s determination, which permitted the use of the deidentified existing data under the approved protocol.

#### Privacy and Confidentiality

All data used in this study were fully deidentified before analysis. No identifiable information was accessed or included at any stage of this study, ensuring participant privacy and confidentiality in compliance with ethical guidelines.

#### Compensation

No compensation was provided to participants as this study used existing data from a health care program and did not involve any direct participant engagement.

#### Use of Images or Identifiable Data

No images or other materials that could identify individual participants are included in this paper or multimedia appendixes. Thus, there are no concerns regarding identification, and no additional consent for images was required.

## Results

### Summary Statistics

The summary statistics are presented in Table 2. For the intervention group, 427 patients were included in the 6-month analysis, and 338 patients were included in the 12-month analysis. The difference is due to patients with missing BP readings. However, the demographics and the BP status in the first month were similar between the sample of patients at the 6-month and 12-month marks, suggesting that missing BP readings are not correlated with these factors. Compared to the usual care group, for both the 6-month analysis and 12-month analysis, there were fewer females, more Chinese patients, and more patients with controlled first-month BP in the PTEC-HT group. The patients in the PTEC-HT group were younger compared to the usual care group.

**Table 2:** Summary statistics.

	6-month analysis				12-month analysis		
	PTEC-HT^a^	Usual care	*P* value	PTEC-HT		Usual care	*P* value
Number of observations	427	64,679	N/A^b^	338		7324	N/A
Gender: female (vs male), n (%)	183 (42.9)	34,826 (53.8)	<.001	149 (44.1)		3827 (52.2)	<.001
Ethnicity: Chinese (vs non-Chinese), n (%)	387 (90.6)	52,619 (81.4)	<.001	308 (91.1)		5821 (79.5)	<.001
Age (years), mean (SD)	58.8 (10.1)	64.7 (11.6)	<.001	58.9 (9.9)		65.6 (11.8)	<.001
Controlled BP^c,d^ at first month, n (%)	339 (79.4)	44,751 (69.2)	<.001	273 (80.8)		4897 (66.9)	<.001
Controlled BP at sixth month, n (%)	384 (89.9)	47,246 (73)	<.001	N/A		N/A	N/A
Controlled BP at 12th month, n (%)	N/A	N/A	N/A	309 (91.4)		5274 (72)	<.001
Improved BP first month to sixth month, n (%)	191 (44.7)	21,154 (32.7)	<.001	150 (44.4)		2461 (33.6)	<.001
Improved BP seventh month to 12th month, n (%)	N/A	N/A	N/A	102 (30.2)		2309 (31.5)	.64
Improved BP first month to 12th month, n (%)	N/A	N/A	N/A	157 (46.4)		2540 (34.7)	<.001

^a^PTEC-HT: Primary Technology-Enhanced Care for Hypertension.

^b^N/A: not applicable.

^c^BP: blood pressure.

^d^A patient was defined as having controlled blood pressure in a month if the mean systolic blood pressure <140 and mean diastolic blood pressure <90. A patient was defined with improved blood pressure if systolic blood pressure and diastolic blood pressure decreased.

### BP Improvement

In the 6-month analysis and 12-month analysis, a higher proportion of patients had improved BP from the first month to the sixth month in the PTEC-HT group compared to the usual care group. In the 12-month analysis, a similar proportion of patients had improved BP from the seventh month to the 12th month between the PTEC-HT group and usual care group. This indicates that the improvement in BP status mainly occurred during the first 6 months of the intervention.

### Direct Medical Cost

Table 3 shows the breakdown of the direct medical cost. For the 6-month analysis, the savings were from doctor consultation, laboratory services, and medication. The total saving was S $73.38. The savings from doctor consultations were the highest, at S $46.04. For the 12-month analysis, the total saving was S $209.09, which was almost 3 times the savings for the 6-month analysis. This was mainly driven by the additional savings from doctor consultations. The savings from doctor consultation was S $159.76 at the 12-month analysis. The reduction in doctor consultation cost was consistent with the number of face-to-face visits observed. Over the first 6 months, patients from the PTEC-HT group had 0.8 fewer face-to-face visits compared to the patients from the usual care group. Over the 12 months, patients from the PTEC-HT group had 2.8 fewer face-to-face visits compared to the patients from the usual care group. The cost of nurse services was higher for the PTEC-HT group compared to the usual care group for both the 6-month analysis and 12-month analysis.

**Table 3:** Direct medical cost^a^.

		Doctor	Nurse	Laboratory	Medication^b^	Total
**Cost per patient at 6 months (S $)**						
	PTEC-HT^c^	98.64	16.68	30.71	130.61	277.46
	Usual care	144.68	9.2	41.98	154.05	350.84
	Difference (PTEC-HT—usual care)	–46.04	7.47	–11.27	–23.44	–73.38
	*P* value	<.001	<.001	<.001	<.001	<.001
**Cost per patient at 12 months (S $)**						
	PTEC-HT	173.03	29.35	70.58	253.02	527.43
	Usual care	332.79	16.37	76.55	309.18	736.52
	Difference (PTEC-HT—usual care)	–159.76	12.97	–5.96	–56.16	–209.09
	*P* value	<.001	<.001	.04	<.001	<.001

^a^A currency exchange rate of S $1=US $0.74 was applicable.

^b^Both hypertension-related and unrelated medication costs were considered. For doctor, nurse, and laboratory costs, only hypertension-related costs were considered.

^c^PTEC-HT: Primary Technology-Enhanced Care for Hypertension.

Table 4 shows the cost of doctors and nurses separately for in-person services and teleconsultation for patients in the PTEC-HT group. For doctor consultations, the cost of teleconsultation was relatively low as the teleconsultation and follow-ups were mainly conducted by nurses. The cost of teleconsultation and follow-ups provided by nurses made up two-thirds of the total cost of services provided by nurses.

**Table 4:** Cost of doctor and nurse service—by face-to-face service and teleservice for PTEC-HT^a^ group^b^.

		Total	Face-to-face service	Teleservice
**Doctor**				
	Cost per patient at 6 months (S $)	98.64	92.3	6.34
	Cost per patient at 12 months (S $)	173.03	163.79	9.24
**Nurse**				
	Cost per patient at 6 months (S $)	16.68	5.28	11.4
	Cost per patient at 12 months (S $)	29.35	10.51	18.84

^a^PTEC-HT: Primary Technology-Enhanced Care for Hypertension.

^b^A currency exchange rate of S $1=US $0.74 was applicable.

### Regression Results

The regression results are presented in Table 5. For BP status, odds ratios are presented. A value higher than 1 means higher odds of having controlled BP status. The coefficient of interest is the coefficient for the interaction term between the intervention group and the postintervention dummy variable. For example, in the 6-month analysis, the value of the coefficient was 2.87 (95% CI 1.84-4.49) using matching plus regression, which shows that BP status improved more for the PTEC-HT group compared to the usual care group at the 6-month mark. For direct medical costs, the original coefficient was presented. The value of the coefficient is not intuitive to interpret; however, the sign of the coefficient (positive or negative) shows whether the variable is associated with a higher or lower cost. A positive coefficient means a higher cost. The coefficient of interest is the coefficient for the intervention group. For example, in the 6-month analysis, the value of the coefficient was –0.19 (95% CI –0.20 to –0.18) using matching plus regression, which shows that the direct medical cost was lower in the PTEC-HT group compared to the usual care group.

The marginal effects are presented in Table 6. For BP status, the value shows the difference in probabilities of having controlled BP between the PTEC-HT group and usual care group, which can be interpreted as an incremental health benefit. For example, in the 6-month analysis, the value was 13.5% (95% CI 6.3%-20.7%) using matching plus regression. This means that the probability of having controlled BP is 13.5% higher for the PTEC-HT group compared to the usual care group. For direct medical cost, the value shows the difference in cost per patient between the PTEC-HT group and the usual care group, which can be interpreted as an incremental cost. For example, in the 6-month analysis, the value was –S $57.7 (95% CI –61.0 to –54.4) using matching plus regression. This means the cost per patient is S $57.7 lower in the intervention group compared to the usual care group. Overall, the PTEC-HT group had better BP status and lower direct medical costs. The intervention is cost-saving. The probability of the intervention being cost-saving will be high given that none of the 95% CIs intercept 0. Compared to using regression alone, matching plus regression gave higher incremental health benefits and lower cost savings.

The total cost includes direct medical cost, cost of Bluetooth-enabled BP machine, and program maintaining cost, including data storage and app maintenance.

**Table 5:** Regression results.

		6-month analysis						12-month analysis					
		Regression, coefficient (95% CI)			Matching + regression, coefficient (95% CI)			Regression coefficient, (95% CI)			Matching + regression, coefficient (95% CI)		
**Outcome variable: controlled BP^a^ status^b^**													
	Intervention group * postintervention			1.92 (1.35 to 2.73)			2.87 (1.84 to 4.49)			1.99 (1.29 to 3.06)			3.58 (2.29 to 5.6)
	Intervention group			1.63 (1.29 to 2.07)			1 (0.71 to 1.4)			1.98 (1.5 to 2.61)			1.01 (0.76 to 1.35)
	Postintervention			1.21 (1.18 to 1.23)			0.84 (0.63 to 1.12)			1.28 (1.2 to 1.36)			0.71 (0.65 to 0.79)
	Age			0.99 (0.99 to 0.99)			1.03 (1.02 to 1.05)			1 (0.99 to 1)			1.01 (1 to 1.02)
	Female			0.94 (0.91 to 0.96)			1.18 (0.89 to 1.56)			0.98 (0.91 to 1.06)			1.11 (0.99 to 1.24)
	Chinese			1.27 (1.23 to 1.31)			2.26 (1.5 to 3.41)			1.25 (1.13 to 1.37)			1.77 (1.47 to 2.14)
**Outcome variable: direct medical cost^c^**													
	Intervention group		–0.19 (–0.24 to –0.14)			–0.19 (–0.2 to –0.18)			–0.27 (–0.33 to –0.2)			–0.28 (–0.31 to –0.25)	
	Age		0.007 (0.006 to 0.007)			0.002 (0.001 to 0.002)			0.007 (0.006 to 0.008)			0.003 (0.001 to 0.004)	
	Female		0.07 (0.06 to 0.08)			0.11 (0.1 to 0.12)			0.09 (0.07 to 0.12)			0.12 (0.09 to 0.15)	
	Chinese		–0.11 (–0.12 to –0.1)			–0.022 (–0.04 to –0.003)			–0.13 (–0.16 to –0.09)			0.05 (–0.007 to 0.1)	

^a^BP: blood pressure.

^b^Odds ratios from the regression results are presented.

^c^Original coefficients from the regression results are presented.

**Table 6:** Regression results—marginal effects^a^.

		Probability of controlled BP^b^ status: PTEC-HT^c^ group versus usual care group (%), coefficient (95% CI)			Change in direct medical cost: PTEC-HT group versus usual care group (S $), coefficient (95% CI)			Change in total cost: PTEC-HT group versus usual care group (S $), coefficient (95% CI)					
**6-month analysis**													
	Regression		7 (2.1 to 12)			–65.5 (–83.5 to –47.4)			–6.4 (–24.4 to 11.8)				
	Matching + regression		13.5 (6.3 to 20.7)			–57.7 (–61 to –54.4)			1.45 (–1.85 to 4.75)				
**12-month analysis**													
	Regression			5.9 (0.3 to 11.4)			–193.6 (–238.5 to –148.7)			–75.3 (–120.2 to –30.4)			
	Matching + regression			16 (10.7 to 21.3)			–170.9 (–189.9 to –151.9)			–52.6 (–71.6 to –33.6)			

^a^A currency exchange rate of S $1=US $0.74 was applicable.

^b^BP: blood pressure.

^c^PTEC-HT: Primary Technology-Enhanced Care for Hypertension.

### Considering Additional Costs

The Bluetooth-enabled BP machine was S $100. Assuming a lifespan of 3 years and using a 3% discount rate, the yearly cost was S $34.3. Program maintenance cost was S $7 per patient per month. The change in total cost considering these additional costs is presented in Table 6. The PTEC-HT program reached breakeven at around the sixth month. In the 12th month, the program led to savings even after these additional costs were included.

## Discussion

### Comparison With Prior Work

This study examined the effectiveness and cost-effectiveness of the PTEC-HT scaling program, a telehealth intervention currently being implemented nationwide in Singapore for patients with hypertension, using real-world data. Compared to the usual care of patients visiting polyclinics, the PTEC-HT scaling program was found to be cost-saving and more effective in controlling BP status. This agrees with the literature, including the PTEC-HT pilot study, that telemonitoring and teleconsultation are effective in controlling BP status [10,20,21]. However, this study showed that the improvement in BP status mainly happened during the first 6 months. From the seventh month to the 12th month, the intervention mainly helped the patients maintain their BP status. This raises the question of whether the intervention should be stopped at the end of the sixth month. In our study, the savings in direct medical cost continued beyond the first 6 months, which supports that the intervention should continue beyond the sixth month. Furthermore, the patients’ BP status may have reverted if the intervention ended in the sixth month. Currently, the PTEC-HT program is still ongoing. Data at the 18th month and 24th month will be collected to understand whether it is worthwhile to continue the intervention for 2 years.

While patients’ BP status was better, the medication cost was lower in the PTEC-HT group compared to the usual care group. With better BP control, doctors up-titrated BP medications less. This also led to fewer serum electrolyte tests, which are needed when the dose of BP medications such as angiotensin-converting enzyme inhibitors and angiotensin receptor blockers are increased. Furthermore, with e-nudging, patients self-monitored their BP more regularly. As a result, patients learned how their BP was better controlled with medication adherence, so they took their antihypertensives more regularly. They also probably adhered better to their salt restriction and regular exercise as this was a part of the education that they received through their PTEC mobile app. Future studies should be taken to examine the details of medication consumption and adherence.

In the PTEC-HT pilot study, in the sixth month, while the probability of having controlled BP status was 18.9% higher in the intervention group compared to the usual care group, the cost was S $88.56 higher in the intervention group [10]. Compared to the PTEC-HT pilot study, the PTEC-HT scaling program was relatively less effective in improving BP status but at a lower cost. However, the PTEC-HT program in the scaling phase continued to be more effective than the usual care group. A possible explanation of differences in the pilot and scaling phase may be related to the PTEC-HT pilot study being an experimental study and the PTEC-HT scaling-up study being a real-world implementation program. In the experimental setting, patients may be more adherent to the intervention compared to the patients in the real-world setting. The researchers and health care providers may also implement the intervention more carefully to demonstrate the concept and “efficacy” of the intervention in a controlled setting. However, real-world implementation of the intervention shifts the focus to a more pragmatic approach, testing the “effectiveness” of the intervention in the usual care setting with a more heterogeneous group of patients and associated parameters. This transition between the explanatory and pragmatic approaches resulting in a difference in the magnitude of the effect estimates is well documented in existing literature [22-24]. Hence, a higher effect in the PTEC-HT pilot compared to PTEC-HT scaling up should be expected. However, at the same time, in the experimental setting, researchers and health care providers may be more conservative, being unfamiliar with the new intervention and hence may have provided more services than required, which could lead to higher costs. Our study illustrates the transition gap in effectiveness and cost between the experimental and real-world setting, highlighting the importance of adopting a phased approach to implementing an intervention and undertaking continuous evaluation alongside scaling efforts to support subsequent real-world policy change.

### Program Setup Cost

The program setup cost for teleintervention could be high and may act as one barrier preventing the adoption of the program. This information was not reported in this paper as it is confidential. In general, we believe that the program setup cost will not change the main conclusion of this study. First, most of the program setup costs are 1-time costs. As more patients sign up for the program, the cost per patient will be lower. Second, many teleinterventions can share the hardware and system, which can further reduce the cost per program. Currently, there are other ongoing PTEC interventions, such as a telemonitoring program for patients with diabetes [25]. Third, Singapore is a high-income country, and digitalization is one of its policy priorities. The program setup cost is unlikely to act as a barrier to program adoption in Singapore. Nevertheless, the program setup cost could be a barrier for resource-poor settings, which should be considered by researchers when evaluating similar interventions in resource-poor settings. To assess the impact of the program setup cost, we estimated the time needed to recover the initial investment if the intervention were scaled to the national level. Assuming the prevalence of doctor-diagnosed hypertension in Singapore residents aged 18-74 years as being 21% to 22%, with 50% of these patients with hypertension seeking care in public polyclinics, that 20% of these have simple hypertension, that the uptake rate of PTEC-HT program is between 20% to 40%, and taking the cost saving range of S $52 per patient per year to S $75 per patient per year, the duration of PTEC-HT initial set up cost recovery period is from less than a year to up to 2.5 years.

### Inflation

In our analysis, the cost was converted to the year 2020, when the PTEC-HT scaling-up trial started. However, the results remain relevant to inform current decision-making. From 2020 to 2023, the inflation rate for the health care sector in Singapore was 8% [26]. Considering inflation and adjusting the cost to the year 2023 will further increase savings from the PTEC-HT compared to usual care. Moreover, as the conclusions related to the relative difference between PTEC-HT and usual care remain unchanged (after including the inflation impact) with PTEC-HT being cost saving when compared to usual care, the findings from the current analysis are being used to inform policy decisions related to the future funding of the program, sustainability considerations, and making it part of the usual care in the public primary care setting of Singapore. At the same time, it is important to continuously monitor and evaluate the effectiveness and cost-effectiveness of the program in the long run.

### Challenges in Using Real-World Evidence

Worldwide, there is a growing interest in using real-world data to inform regulatory and reimbursement decisions. Several challenges exist when using real-world data and need to be dealt with [27,28]. First, data quality varies in the real-world setting. Low-quality data could render the results obtained invalid. In our study, the use and cost data of patients in the intervention group were prospectively collected and stored in the electronic medical record system in the polyclinics. The medical and use data of patients in the usual care group were routinely collected retrospective data from the polyclinics and stored in the national claims database. As this national claims database is routinely used for policy-related analysis and the evaluation of different programs, the quality of the data can be considered high in our study.

Second, the analytical method used is critical to account for potential bias. Unlike experimental studies, patients are unlikely to be randomly assigned to the intervention group and the usual care group in a real-world setting. Systematic differences between groups could exist, which may introduce bias. Econometric methods can be used to address the issue. In our study, participants from the PTEC-HT group measured their BP at home, and participants from the usual care group measured their BP in the clinics. The difference in BP outcomes between the home setting and the clinic setting has been documented in the literature as the “white coat effect” [29,30]. Leveraging panel data, we addressed this issue by comparing the change in BP status over time between the PTEC-HT group and the usual care group. Our approach, which resembles the difference-in-differences method, effectively removes the fixed effect, including biases introduced by the measurement setting. Besides regression analysis adjusting for confounders, matching was also used. We further tested the robustness of our results using quantile regression and removing patients with extremely high costs. Our results are robust to different methods. Hence, our study also contributes to the literature on using real-world data to analyze public health interventions.

### Limitations

There are several limitations to this study. First, the confounding impact of SARS-COV-2 cannot be removed. The data for the usual care group were in the years 2018 and 2019 before SARS-COV-2. The data for the PTEC-HT group were after SARS-COV-2. The intervention started in August 2020 when patients were allowed to visit polyclinics to manage their conditions under relatively less stringent social distancing measures. Hence, the impact of SARS-COV-2 on the intervention is expected to be small. Second, the medication cost cannot be separated into hypertension related and non–hypertension related. In our analysis, we considered the medication cost of all drugs. The intervention is not expected to affect the cost of non–hypertension-related drugs. Hence, the difference in medication cost should reflect the difference due to hypertension-related drugs. Third, long-term health outcomes were not considered. The improvement in BP status could result in a reduced chance of cardiovascular complications in the long run [31,32]. However, this is out of the scope of this study. Including the longer-term health benefits will further favor the intervention. Fourth, while PTEC-HT was shown to be effective and cost-effective, equity issues should also be considered. Patients from certain subpopulation groups, such as those with low digital health literacy, may benefit less from the intervention [33]. Efforts should be made to help these patients adopt telehealth intervention to ensure the benefit to all. Last, the PTEC-HT intervention empowers nurses to manage patients with hypertension independently. In this study, we extrapolated the time spent by nurses from a similar intervention involving the management of patients with type 2 diabetes to estimate the cost of providing telemanagement services. Accurately capturing the time spent by nurses is essential to assess the potential workload and burden associated with managing the PTEC-HT program. Currently, a time-motion study for the PTEC-HT program is ongoing, which will provide a more precise estimate of nurse-related costs. Nevertheless, the cost-effectiveness results for this study are unlikely to change significantly, as nurse costs constitute a relatively small proportion of the total cost.

### Conclusions

Implemented in a real-world setting in Singapore, our study showed that the PTEC-HT scaling program is more effective in controlling BP status with lower direct medical cost compared to the usual care over both 6 months and 12 months. Our study results support the adoption of the PTEC-HT program (ie, a telemonitoring and teleconsultation intervention) at scale to help patients manage hypertension.

## Appendix

Multimedia Appendix 1 CHEERS (Consolidated Health Economic Evaluation Reporting Standards) 2022 checklist.

Multimedia Appendix 2 Additional analysis.

## Abbreviations

BP: blood pressure
CHEERS: Consolidated Health Economic Evaluation Reporting Standards
ICD-10: International Classification of Diseases, Tenth Revision
MOHT: Ministry of Health Office for Healthcare Transformation
NHGP: National Healthcare Group Polyclinics
PTEC: Primary Technology-Enhanced Care
PTEC-HT: Primary Technology-Enhanced Care for Hypertension
