# Supplementary material for: Primary Technology-Enhanced Care for Hypertension Scaling Program: Trial-Based Economic Evaluation Examining Effectiveness and Cost-Effectiveness Using Real-World Data in Singapore
Source: J Med Internet Res. 2025 Apr 15;27:e59275. doi: 10.2196/59275 (PMC12041820; doi:10.2196/59275)
Supplement: Multimedia Appendix 2 [file jmir_v27i1e59275_app2.docx]

**Additional Analysis**

Additional analyses were conducted to test the robustness of the results focusing on medical cost. The results are presented in Table S1. For the ease of comparison, marginal effects are presented. Baseline regression results from the main article are presented for comparison.

Remove patients in usual-care group with cost at top 2%

Patients with extremely high medical cost may drive up the results on cost. We removed these patients from the usual-care group. The results obtained from this analysis serve as conservative estimates of the cost saved.

Quantile regression

As medical cost usually does not follow normal distribution, additional insight can be obtained by examining the results at different quantiles. We conducted quantile regression considering 0.75 quantile, 0.5 quantile, and 0.25 quantile.

Subgroup analysis

We did subgroup analysis considering patients with controlled baseline blood pressure and patients with uncontrolled baseline blood pressure. This is to understand whether the medical cost saved from the programme depends on patients’ baseline blood pressure.

Overall, the results are consistent across different methods. One point deserves to highlight is the results from the subgroup analysis. During the first six months, the saving in medical costs was higher from patients with controlled baseline blood pressure compared to patients with uncontrolled baseline blood pressure. Over the twelve-months period, the medical costs saved were similar between patients with controlled baseline blood pressure and patients with uncontrolled baseline blood pressure.

**Table S1**. Additional analysis.

|  | 6^th^-month analysis | | 12^th^-month analysis | |
| --- | --- | --- | --- | --- |
|  | Marginal effect | 95% Confidence Interval | Marginal effect | 95% Confidence Interval |
|  |  |  |  |  |
| **Baseline results** | S$ -65.5 | S$ (-83.5, -47.4) | S$ -193.6 | S$ (-238.5, -148.7) |
| **Remove top 2%** | S$ -52.9 | S$ (-67.6, -38.2) | S$ -171.7 | S$ (-208.3, -135.2) |
| **Quantile regression** |  |  |  |  |
| **0.75 quantile** | S$ -45.3 | S$ (-71.1, -19.6) | S$ -175.9 | S$ (-230.9, -120.9) |
| **0.5 quantile** | S$ -54.7 | S$ (-71.7, -37.6) | S$ -148.1 | S$ (-191.0, -105.3) |
| **0.25 quantile** | S$ -64.5 | S$ (-78.5, -50.5) | S$ -151.2 | S$ (-187.3, -115.1) |
| **Subgroup analysis by baseline blood pressure** |  |  |  |  |
| **Controlled** | S$ -77.0 | S$ (-97.0, -56.9) | S$ -196.8 | S$ (-247.3, -146.3) |
| **Uncontrolled** | S$ -18.5 | S$ (-59.3, 22.4) | S$ -171.9 | S$ (-272.1, -71.7) |

**ICD 10 Code Used to Exclude Patients with Pre-existed Complication**

The ICD 10 codes below were used for patient exclusion for both the PTEC-HT group and the usual-care group:

Pre-diabetes: E09, E0921, E0929, E0931, E0932, E0940, E0942, E0951, E0952, E0971, E0972, E098, E099, O2452 – O2454, O2459

Diabetes Mellitus: Z9222, E10* - E11*, E13* - E14*, E15, O24, O240, O2412 – O2414, O2419, O2422 – O2424, O2429, O2432 – O2434, O2439

Stroke: I60* - I64*

Ischaemic heart disease: I20*, I21*, I22*, I24, I248, I249, I25, I250, I2510-I2513, I252, I255, I256, I258, I259

Nephritis/Nephrosis: N03*, N04*, N06*, N07*, N11, N110-N118, N18*
